# Proteome-Wide Analysis of Lysine 2-Hydroxyisobutyrylated Proteins in *Fusarium oxysporum*

**DOI:** 10.3389/fmicb.2021.623735

**Published:** 2021-02-10

**Authors:** Hengwei Qian, Lulu Wang, Xianliang Ma, Xingling Yi, Baoshan Wang, Wenxing Liang

**Affiliations:** ^1^College of Plant Health and Medicine, Qingdao Agricultural University, Qingdao, China; ^2^College of Life Sciences, Shandong Normal University, Jinan, China; ^3^Micron Biotechnology Co., Ltd., Hangzhou, China

**Keywords:** lysine 2-hydroxyisobutyrylation, post-translational modification, *Fusarium oxysporum*, proteomics, virulence

## Abstract

Protein lysine 2-hydroxyisobutyrylation (K_*hib*_), a new type of post-translational modification, occurs in histones and non-histone proteins and plays an important role in almost all aspects of both eukaryotic and prokaryotic living cells. *Fusarium oxysporum*, a soil-borne fungal pathogen, can cause disease in more than 150 plants. However, little is currently known about the functions of K_*hib*_ in this plant pathogenic fungus. Here, we report a systematic analysis of 2-hydroxyisobutyrylated proteins in *F. oxysporum*. In this study, 3782 K_*hib*_ sites in 1299 proteins were identified in *F. oxysporum*. The bioinformatics analysis showed that 2-hydroxyisobutyrylated proteins are involved in different biological processes and functions and are located in diverse subcellular localizations. The enrichment analysis revealed that K_*hib*_ participates in a variety of pathways, including the ribosome, oxidative phosphorylation, and proteasome pathways. The protein interaction network analysis showed that 2-hydroxyisobutyrylated protein complexes are involved in diverse interactions. Notably, several 2-hydroxyisobutyrylated proteins, including three kinds of protein kinases, were involved in the virulence or conidiation of *F. oxysporum*, suggesting that K_*hib*_ plays regulatory roles in pathogenesis. Moreover, our study shows that there are different K_*hib*_ levels of *F. oxysporum* in conidial and mycelial stages. These findings provide evidence of K_*hib*_ in *F. oxysporum*, an important filamentous plant pathogenic fungus, and serve as a resource for further exploration of the potential functions of K_*hib*_ in *Fusarium* species and other filamentous pathogenic fungi.

## Introduction

Post-translational modifications (PTMs) of proteins play key roles in diverse biological processes of cells and are dynamic and reversible modification reactions occurring during or after biosynthesis, including amino acid synthesis, protein interaction networks, and energy metabolism (Zhou et al., 2016; Liu et al., 2018a). In recent years, with the development of mass spectrometry (MS) (Huang et al., 2014), lysine (K) has been identified as a primary residue for PTMs, including crotonylation (K_*cr*_), acetylation (K_*ac*_), malonylation (K_*ma*_), succinylation (K_*su*_), methylation (K_*me*_), butyrylation (K_*bu*_), and glutarylation (K_*glu*_), which were named lysine acylation (Chen et al., 2007; Peng et al., 2011; Peach et al., 2012; Tan et al., 2014; Li D. et al., 2016; Li Y. et al., 2016; Zhang et al., 2017; Cheng et al., 2020). Several types of lysine acylation occur because lysine is an alkaline amino acid that contains an unstable ε-NH_2_ side chain that can interact with diverse chemical groups (Xu et al., 2017). Many PTMs have been discovered to modulate chromatin packaging by changing the charge of lysine slide chains. In addition, PTMs may regulate enzyme activity or protein structure, further affecting protein functions (Huang et al., 2015).

Recently, lysine 2-hydroxyisobutyrylation (K_*hib*_), a type of lysine modification, was discovered in histones and is conserved from yeast to humans (Dai et al., 2014; Huang et al., 2017, 2018). In a mass spectrometry analysis, the reporter indicated that K_*hib*_ has a mass shift of +86.03 Da because of the ε-NH_2_ side chain of K_*hib*_ (Dai et al., 2014). Recently, K_*hib*_ was identified as a new histone mark in eukaryotic cells, and 63 K_*hib*_ sites on histone proteins have been identified by MS analysis and biochemical methods in humans and mice (Dai et al., 2014; Cheng et al., 2020). In this study, histone H4K8_*hib*_ was found to regulate gene transcriptional activity and was a better indicator of high gene expression than H4K8_*ac*_. In addition, K_*hib*_ exists in the N-termini of histone proteins and the main globular domain, but acetylation mainly occurs in the N-termini of histone proteins. Obviously, 2-hydroxyisobutyrylation structurally differs from acetylation (Dai et al., 2014; Cluntun et al., 2015). A proteome-wide analysis of K_*hib*_ in *Saccharomyces cerevisiae* identified 1458 K_*hib*_ sites on 369 histones and non-histone proteins, and a bioinformatics analysis showed that K_*hib*_ was enriched in the glycolysis/gluconeogenesis pathway. Interestingly, the histone H4K8_*hib*_ was regulated by glucose homeostasis and influenced cell proliferation in *S. cerevisiae* (Huang et al., 2017). In prokaryotic cells, K_*hib*_ is widely distributed, and 4735 K_*hib*_ sites on 1051 proteins were identified in *Proteus mirabilis* by affinity enrichment with two-dimensional liquid chromatography (LC) separation and MS analysis. The most 2-hydroxyisobutyrylated proteins were involved in diverse biological processes based on the bioinformatics analysis; importantly, central metabolism enzymes were found to be 2-hydroxyisobutyrylated in *P. mirabilis* (Dong et al., 2018). In a study of developing rice seeds, which was the first report of K_*hib*_ in plant, 9916 K_*hib*_ sites on 2512 proteins were identified. Functional annotation analyses indicated that 2-hydroxyisobutyrylated lysine is essential for various biological processes, including the TCA cycle, starch biosynthesis, lipid metabolism, and protein biosynthesis (Meng et al., 2017). Using a specific antibody combined with LC-MS/MS, a total of 11 976 K_*hib*_ sites in 3001 proteins were found in *Physcomitrella patens*, an important plant model system used for physiological studies. A systematic analysis of K_*hib*_ sites in *P. patens* histone proteins demonstrated some conserved sites in histone H3 and H4 proteins and revealed unknown sites in histone H1, H2A, and H2B proteins (Vidali and Bezanilla, 2012; Yu et al., 2017).

Histone acetylation and deacetylation of lysine residues are reversible processes and are catalyzed by histone acetyltransferases (HATs) and histone deacetylases (HDACs), respectively (Lv et al., 2016). In addition to acetylation and deacetylation catalysis, HATs and HDACs can also catalyze other acylation or deacylation reactions such as 2-hydroxyisobutyrylation, implying that the functions of new acylations may be similar to or redundant with histone acetylation (Cheng et al., 2009; Sabari et al., 2015). The HAT Esa1p and its human homolog Tip60 could catalyze K_*hib*_ reaction *in vitro* and *in vivo* (Huang et al., 2018). In addition, the HDACs HDAC1, HDAC2, and HDAC3 function as reverse enzymes to remove K_*hib*_ in both *in vitro* and *in vivo* reactions in mammalian cells. The findings of K_*hib*_ transferase and de-2-hydroxyisobutyrylation enzymes greatly increase the knowledge of K_*hib*_ and expand the perspective of protein functions (Dai et al., 2014; Huang et al., 2017).

However, to date, no evidence of K_*hib*_ has been reported in filamentous plant pathogenic fungi. *Fusarium* species are the most diverse and widely dispersed filamentous plant pathogentic fungi in the world that causes the economic loss and reduces the crop yields. Some *Fusarium* species, such as *Fusarium graminearum*, *Fusarium asiaticum*, and *Fusarium verticillioides*, could infect the predominantly the cereals, but the *Fusarium oxysporum* has a broad host range (Ma et al., 2010; Alwahshi et al., 2019). *F. oxysporum* is a soil-borne phytopathogenic fungus that can cause root rot or wilting disease in more than 150 different plants, including tomato, potato, banana, melon, pine, and date palm (*Phoenix canariensis*) (Mohali, 1996; Pietro et al., 2003; Perez-Nadales and Di Pietro, 2011; Lan et al., 2020). At first, the conidia of *F. oxysporum* adhere to the surface of the host plant and then invade and colonize the roots, thereby absorbing nutrients and water, resulting in a reduction in plant growth or even plant cell death (Michielse and Rep, 2009; Rana et al., 2017). Individual *F. oxysporum* strains were defined as forma specialis (f. sp.) based on various hosts, for instance, *F. oxysporum* f. sp. *Lycopersici*, also called *Fol*, is a tomato pathogen (Kashiwa et al., 2016; Rana et al., 2017). K_*hib*_, as a type of PTM, occurs on lysine residues and is expected to play important roles in biological processes and molecular functions in *F. oxysporum*. To test this hypothesis, we performed the first global analysis of K_*hib*_ in *F. oxysporum*. In total, 3782 K_*hib*_ sites in 1299 proteins were identified and involved in various biological processes. The results of a bioinformatics analysis showed that the 2-hydroxyisobutyrylated proteins were localized in multiple cellular compartments, including the cytoplasm, nucleus, mitochondria and plasma membrane, with diverse molecular functions. Importantly, we found that several 2-hydroxyisobutyrylated proteins play a regulatory role in the virulence or conidiation of *F. oxysporum*. This work provides insights into the lysine 2-hydroxyisobutyrylome in *F. oxysporum* and serves as a dataset for exploring the function of 2-hydroxyisobutyrylated proteins in this pathogen.

## Materials and Methods

### Fungal Strain and Culture

The *F. oxysporum* f. sp. *lycopersici* strain 4287 was used in this study (Ma et al., 2010). The *F. oxysporum* was cultured in potato Dextrose agar (PDA) (Solarbio, Beijing, China) at 25°C for 3 days, and then was taken from the colony and transferred into PDB (Potato Dextrose Broth) medium to produce conidia. The conidia were harvested and incubated in YEPD medium at 25°C with shaking at 180 rpm for 14 h. The harvested mycelia were immediately frozen in liquid nitrogen and stored at −80°C.

### Protein Extraction and Trypsin Digestion

The mycelia were ground into powder in liquid nitrogen (Zhou et al., 2016; Liu et al., 2018a). Next, the powder sample was suspended in 5 mL lysis buffer containing 8 M urea, 1% Triton X-100, 65 mM dithiothreitol (DTT), 0.1% protease inhibitor cocktail, 50 mM nicotinamide, 2 mM EDTA, and 3 μM Trichostatin A (Solarbio, Beijing, China) and then sonicated for three times on ice using a high intensity ultrasonic processor (Scienta, Ningbo, China) (Lv et al., 2016). The cell debris was separated by centrifugation at 15 000 × *g* and 4°C for 15 min, and the proteins were precipitated with 15% cold TCA (Sigma, Darmstadt, Germany) at 4°C for 2 h. After centrifugation at 4°C for 15 min, the supernatant was discarded, and the remaining protein was washed three times with cold acetone (Yuandong, Yantai, China). Finally, the target protein was redissolved in 8 M urea supplemented with 100 mM (NH_4_)_2_CO_3_ (PH 8.0) and the protein concentration was determined with 2-D Quant kit (GE, Fairfield, CT, United States) according to the manufacturer’s instructions. The protein solution was reduced with 5 mM DTT (Solarbio, Beijing, China) at 37°C for 1 h and alkylated with 30 mM iodoacetamide (IAA) (Solarbio, Beijing, China) for 45 min at 25°C in darkness. For digestion, the protein was diluted with 100 mM (NH_4_)_2_CO_3_ to reduce urea concentration. Trypsin (Thermo Fisher Scientific, Waltham, MA, United States) was added into the reaction overnight at 1:50 trypsin-to-protein mass ratio (Liu et al., 2018b). In order to ensure digested completely, trypsin was added again into reaction at 1:100 trypsin-to-protein mass ratio and the mixture reaction was incubated for other 4 h.

### HPLC Fractionation

The sample was separated into fractions by high pH reverse-phase HPLC (Shimadzu, Kyoto, Japan) using Agilent 300 Extend C18 column (5 μM particles, 4.6 mm ID, and 250 mm length). The peptides were separated firstly into 80 fractions with a gradient of 2 to 60% acetonitrile (Solarbio, Beijing, China) in 10 mM (NH_4_)_2_CO_3_ (pH 10.0). Then, the peptides were combined into eight fractions and dried by vacuum centrifuging (Meng et al., 2017; Yin et al., 2019).

### Affinity Enrichment of Lysine 2-Hydroxyisobutyrylated Peptides

For K_*hib*_ peptides enrichment, the tryptic peptides were dissolved in NETN buffer (50 mM Tris–HCl, 100 mM NaCl, 1 mM EDTA, 0.5% NP-40, pH 8.0) and then separated into several fractions. Each fraction was incubated with pre-washed agarose beads conjugated with 2-hydroxyisobutyryllysine antibody (PTM-801) (PTM Biolabs, Hangzhou, China) overnight at 4°C with gentle shaking. Then the beads were washed three times with NETN buffer and twice with cold ddH_2_O. The K_*hib*_ peptides bound to the beads were eluted with 0.1% trifluoroacetic acid (TFA) and then rinsed with C18 Zip Tips (Millipore, Burlington, MA, United States).

### LC-MS/MS Analysis

The K_*hib*_ peptides were reconstituted in 0.1% formic acid (FA) and loaded on a reversed-phase pre-column (Acclaim PepMap 100 C18 column) (Thermo Fisher Scientific, Waltham, MA, United States), and then separated using a reversed-phase analytical column (Acclaim PepMap RSLC C18 column) (Thermo Fisher Scientific, Waltham, MA, United States) on UPLC system. The gradient was composed of an increase from 2 to 10% solvent (0.1% formic acid in 98% acetonitrile) for 6 min, 10 to 20% for 45 min, 20% climbing to 80% in 7 min and then holding at 80% at least for 4 min, all maintaining a flow rate of 250 nl/min. The peptides were subjected to ESI/NSI sources followed by MS/MS in Q ExactiveTM Plus (Thermo Fisher Scientific, Waltham, MA, United States) coupled online to UPLC. Whole peptides and ion fragments were detected in the Orbitrap at a resolution of 70 000 and 17 500, respectively, with NCE setting at 30. The electrospray voltage was set at 2.0 kV to analyze. In order to generate MS/MS spectra, the automatic gain control (AGC) was used to prevent overfilling of the ion trap. The m/z range was from 350 to 1800 for MS scans. The MS fixed first mass was set at 100 m/z.

### Database Cearch

MaxQuant integrated with Andromeda search engine (v.1.5.1.8) was used to analyze the raw data of MS/MS. The tandem mass spectra collected were searched against UniProt *F. oxysporum* f. sp. *lycopersici* database (17 735 sequences) concatenated with reverse decoy database. Mass errors of precursor and fragment ions were set as 10 ppm and 0.02 Da, respectively. Trypsin/P was specified as cleavage enzyme allowing up to four missing cleavage, five modifications per peptide and five charges. Carbamidomethylation on Cysteine was specified as fixed modification. Oxidation of methionine and 2-hydroxyisobutyrylation both on lysine and protein N-terminal were fixed as variable modifications. The minimal peptide was set to seven, and the false discovery rate (FDR) threshold for modification sites and peptides were set as 1%. The K_*hib*_ site localization probability of <0.75 was excluded.

### Bioinformatics Analysis

Gene ontology (GO) of 2-hydroxyisobutyrylation proteome was derived from the UniProt-GOA database^1^. Firstly, converting the identified protein ID to UniProt ID and then mapping to GO IDs by protein ID. If the identified proteins were not annotated by UniProt-GOA database, the InterProScan soft would be used to annotate protein’s GO functional based on protein sequence alignment method (Jiao et al., 2012). Then proteins were classified by GO annotation based on three categories: biological process, cellular component, and molecular function. The software WoLF PSORT was used to predict the subcellular localization of the 2-hydroxyisobutyrylated proteins. Protein secondary structures (α-helix, β-strand, and coil) were analyzed by the online tool NetSurfP (Klausen et al., 2019). Soft MoMo (motif-x algorithm) was used to analyze the sequences model of 2-hydroxyisobutyrylated proteins constituted with amino acids in specific positions of modify-21-mers (10 amino acids upstream and downstream of the site) in all protein sequences (Chou and Schwartz, 2011). Minimum number of occurrences was set to 20. To define the evolutionary conservation of 2-hydroxyisobutyrylation, the BLASTP was used to compare the 2-hydroxyisobutyrylated protein sequences of *F. oxysporum* with *Homo sapiens*, *P. patens*, *Oryza sativa*, *S. Cerevisiae*, and *Toxoplasma gondii*. InterProScan was used to annotate functional description of protein domains based on protein sequences alignment method and the InterPro domain database^2^. Kyoto Encyclopedia of Genes and Genomes (KEGG) database was used to annotate protein pathway description. A two-tailed Fisher’s exact test was used to verify the enrichment of lysine 2-hydroxyisobutyrylated proteins against all database proteins. The protein–protein interaction networks for the 2-hydroxyisobutyrylated proteins were analyzed by using STRING database and visualized in Cytoscape. All projects with a corrected *p*-value < 0.05 is considered significant.

### Generation of the FoFGB1-GFP Strains of *F. oxysporum* and Western Blot Analysis

The coding domain sequence (CDS) of *FoFGB1* was cloned into pYF11-GFP overexpression vector to construct FoFGB1-GFP strains using protoplast transformation of *F. oxysporum*. Total proteins were extracted from conidia and mycelia using lysis buffer (25 mM Tris–HCl pH 8.0, 150 mM NaCl, 0.5 mM EDTA, 0.5% NP-40, 0.5% Triton X-100, 5% glycerol). The protein concentration was determined by BCA protein assay kit (PC0020) (Solarbio, Beijing, China) using BSA as the protein standard. To purify the fusion protein FoFGB1-GFP, 5 mg of total protein was incubated with 25 μL (bead volume) of anti-GFP agarose beads (KT, Shenzhen, China) according the manufacturer’s instructions at 4°C for 3 h. After centrifugation, the beads were washed three times with 1 mL washing buffer (50 mM Tris–HCl pH 7.5, 150 mM NaCl, 0.5 mM EDTA), and the target proteins were eluted completely. The proteins were separated on 12% SDS-PAGE and subjected to immunoblotting using anti-K_*hib*_ (1:5 000 dilution) (PTM-801) (PTM Biolabs, Hangzhou, China) and anti-GFP antibodies (1:10 000 dilution) (ab290) (Abcam, Cambridgeshire, United Kingdom), respectively.

## Results

### Identification of Lysine 2-Hydroxyisobutyrylated Proteins in *F. oxysporum*

Three repeated experiments for identifying 2-hydroxyisobutyrylated proteins and sites were carried out by using a specific antibody and LC-MS/MS analysis, resulting in 5917 sites in 1616 proteins, 6047 sites in 1653 proteins and 5931 sites in 1648 proteins (Supplementary Table 1), respectively. In total, 3782 lysine 2-hydroxyisobutyrylated sites in 1299 proteins were identified from all three replicates (Supplementary Figure 1B and Supplementary Table 2), which account for 7.3% (1299/17 735) of total proteins in *F. oxysporum*. The number of identified 2-hydroxyisobutyrylated proteins in *F. oxysporum* was less than that in rice seed (*O. sativa*) (Meng et al., 2017) and *P. patens* (Yu et al., 2017) but more than that in *S. cerevisiae* (Huang et al., 2017) and *P. mirabilis* (Dong et al., 2018). Together with the identification of 2-hydroxyisobutyrylated proteins in other species, K_*hib*_ is suggested to be a widespread PTM, and this research is the first to report K_*hib*_ in *F. oxysporum*.

### Analysis of 2-Hydroxyisobutyrylated Sites

**FIGURE 1:**
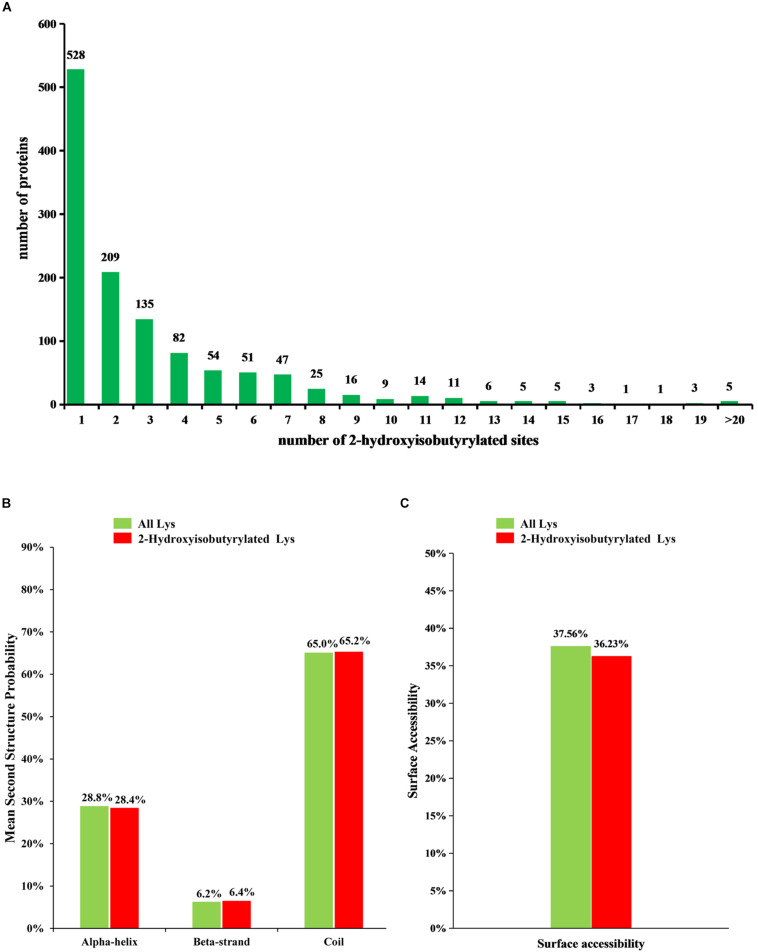
Analysis of 2-hydroxyisobutyrylated sites in *Fusarium oxysporum*. **(A)** Distribution of K_*hib*_ sites in the 2-hydroxyisobutyrylated proteins. The *x*-axis indicates the number of K_*hib*_ sites in protein, whereas the *y*-axis means the proteins number which containing the K_*hib*_ sites. **(B)** Probabilities of lysine 2-hydroxyisobutyrylation in different protein secondary structures (alpha-helix, beta-strand and coil). **(C)** Predicted surface accessibility of 2-hydroxyisobutyrylated lysine was compared with all lysine in *F. oxysporum*.

To understand the distribution of 2-hydroxyisobutyrylated sites, the number of modified sites in each identified protein was calculated in *F. oxysporum*. The result shows that each protein has one or more modified sites, as shown in Figure 1A. From the results, we found that 43.6% of the 2-hydroxyisobutyrylated proteins have only one 2-hydroxyisobutyrylated site and that 56.4% of them contain two or more modified sites.

To determine the relationship between K_*hib*_ and the protein structure in *F. oxysporum*, an analysis of the secondary structure of 2-hydroxyisobutyrylated proteins was performed using the NetSurfP program. The results showed that approximately 34.8% of 2-hydroxyisobutyrylated sites were located in regions with ordered secondary structure, including 28.4% of them located in alpha-helices and 6.4% in beta-strands; however, the 65.2% of 2-hydroxyisobutyrylated sites were distributed in disordered and random coil regions. Furthermore, 2-hydroxyisobutyrylated sites tended to be located in disordered regions when comparing the similarity of the distribution pattern between 2-hydroxyisobutyrylated lysines and all lysines in *F. oxysporum* proteins (Figure 1B), and this result was similar to that from a previous study (Meng et al., 2017). It is suggested that modified 2-hydroxyisobutyrylated lysines are easily located in a folded polypeptide chain because of the flexibility of the disordered regions, and this distribution pattern is similar to that found in *O. sativa*. In addition, the results of the surface accessibility of 2-hydroxyisobutyrylated lysine showed that 36.23% of the modified lysine sites and 37.56% of all residues were exposed to the protein surface (Figure 1C), indicating that K_*hib*_ could slightly affect the surface accessibility of 2-hydroxyisobutyrylated proteins in *F. oxysporum*.

**FIGURE 2:**
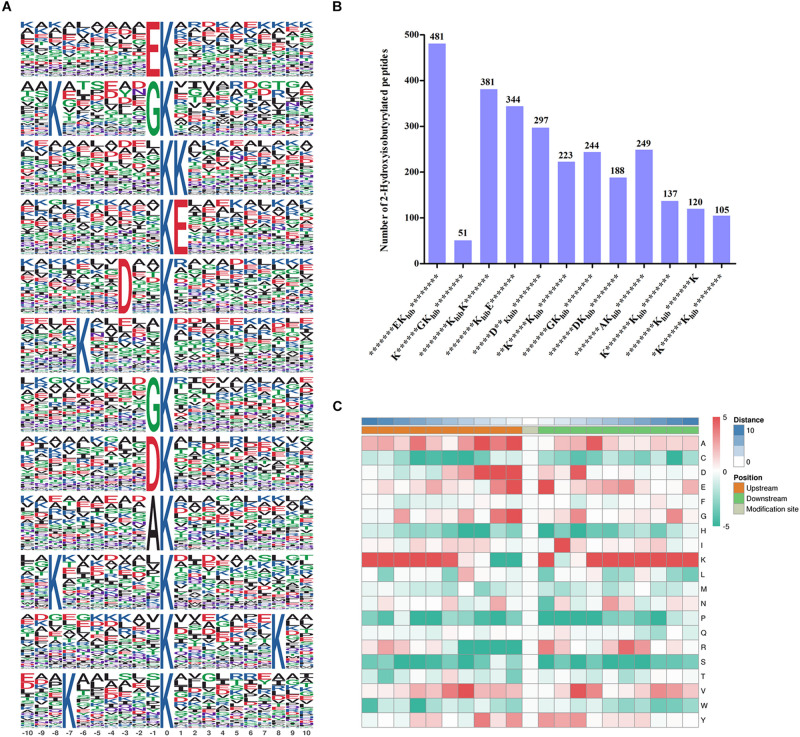
Properties of 2-hydroxyisobutyrylated peptides in *Fusarium oxysporum*. **(A)** 2-hydroxyisobutyrylated motifs and conserved motifs around the 2-hydroxyisobutyrylated sites. **(B)** Frequency of identified 2-hydroxyisobutyrylated peptides in each motif. The ordinate indicates the number of conserved motifs in *x*-axis and the numbers of peptides in different motifs were displayed on top of the columns. K_*hib*_ represents 2-hydroxyisobutyrylated lysine, and * represents a random amino acid residue. **(C)** Heat map of the amino acid compositions of the 2-hydroxyisobutyrylated sites. The middle square represents the K_*hib*_ sites, left and right squares represent the upstream and downstream residues of K_*hib*_ sites, respectively. The red indicates high frequency and green indicates low frequency.

To better identify the amino acid sequences around the 2-hydroxyisobutyrylated lysine sites in *F. oxysporum*, the frequency of motifs in all identified K_*hib*_ peptides were examined by the Motif-x tool. A total of 12 motifs were identified from 2820 K_*hib*_ peptides, which contain sequences from the −10 to +10 position around the 2-hydroxyisobutyrylated lysine (Figure 2A). The different motifs exhibited diverse proportions, and the EK_*hib*_, KK_*hib*_, and K_*hib*_E motifs had large proportions. There were 481, 381, and 344 K_*hib*_ peptides with these motifs, accounting for 17.1, 13.5, and 12.2% of all K_*hib*_ peptides, respectively (Figure 2B). From the results, we can learn that the amino acids with negatively charged side chains, aspartic acid (D), and glutamic acid (E), have a high tendency to be located around the 2-hydroxyisobutyrylated lysine sites. Consistent with this finding, the motifs EK_*hib*_, K_*hib*_E, and DK_*hib*_ were also found in other species, including rice, indicating that K_*hib*_ is a conserved PTM in different species. As shown in the heat map of the amino acid compositions around the 2-hydroxyisobutyrylated sites, the frequencies of arginine (R) at positions −4 to −1 and S (serine) at positions −10 to +10 were the lowest. In addition to D and E, lysine (K) at the −10 to −5 and +4 to +10 positions, valine (V) at the +3 positions and glycine (G) at the −1 position were preferred sites for K_*hib*_ (Figure 2C and Supplementary Table 3).

### Conservation Analysis of 2-Hydroxyisobutyrylated Proteins

**FIGURE 3:**
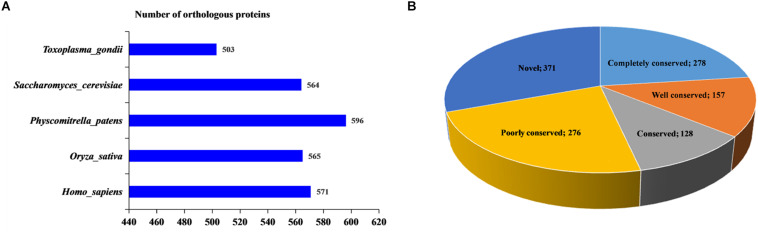
Conservation analysis of 2-hydroxyisobutyrylated proteins in *Fusarium oxysporum* compared with other species. **(A)** Number of orthologous 2-hydroxyisobutyrylated proteins in *Homo sapiens*, *Physcomitrella patens*, *Oryza sativa*, *Saccharomyces cerevisiae*, and *Toxoplasma gondii* with their reported 2-hydroxyisobutyrylomes. The horizontal axis represents the number of orthologous proteins in the species. **(B)** A pie chart of conservation of 2-hydroxyisobutyrylated proteins in five organisms. Grouping was performed as follows: Completely conserved, 5 orthologs; Well conserved, 4 orthologs; Conserved, 3 orthologs; Poorly conserved, 1 to 2 orthologs; and Novel, 0 orthologs.

In this report, using BLASTP, the orthologous 2-hydroxyisobutyrylated protein sequences in *F. oxysporum* were searched against five organisms: *H. sapiens*, *P. patens*, *O. sativa*, *S. cerevisiae*, and *T. gondii*. In total, 2799 orthologs of the 2-hydroxyisobutyrylated proteins in *F. oxysporum* were identified in these five organisms (Figure 3A and Supplementary Table 4). The results showed that 839 2-hydroxyisobutyrylated proteins have orthologs in *T. gondii*, *S. cerevisiae*, *P. patens*, *O. sativa*, and *H. sapiens* and that the numbers of proteins were 503, 564, 596, 565, and 571, respectively, accounting for 69.3% (839/1210) of the total 2-hydroxyisobutyrylated proteins in *F. oxysporum*. Figure 3B shows the conservation of 2-hydroxyisobutyrylated proteins in *F. oxysporum* depending on the number of orthologous proteins in the other five organisms. The pie chart shows that the proportion of completely conserved proteins (with orthologs in all five organisms) was 23.0% (278/1210 proteins). Well-conserved proteins (with orthologs in four organisms) accounted for 13.0% (157/1210 proteins); conserved proteins (with orthologs in three organisms) and poorly conserved proteins (with orthologs in 1 to 2 organisms) accounted for 10.6% (128/1210 proteins) and 22.8% (276/1210 proteins), respectively. In addition, the percentage of novel proteins was 30.7% (371/1210 proteins), these 2-hydroxyisobutyrylated proteins in *F. oxysporum* did not have an ortholog in any of the other five organisms. According to these results, we found that K_*hib*_ is widely conserved in prokaryotes and eukaryotes, but unique 2-hydroxyisobutyrylated proteins are observed in different organisms or species.

### Function Classification and Subcellular Location Analysis of 2-Hydroxyisobutyrylated Proteins in *F. oxysporum*

**FIGURE 4:**
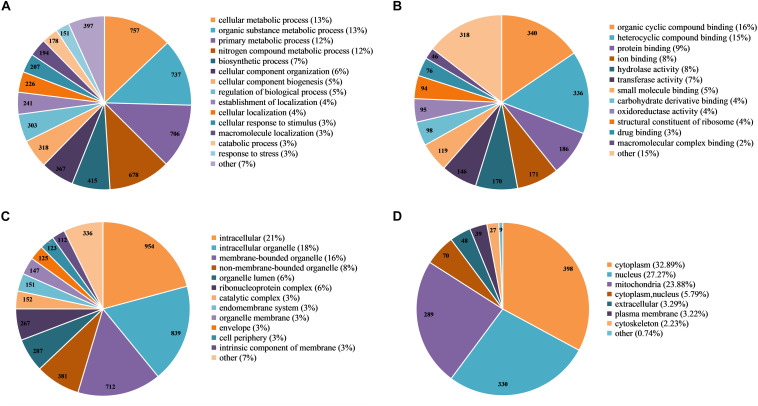
Functional classification of 2-hydroxyisobutyrylated proteins in *Fusarium oxysporum*. **(A)** Classification of the 2-hydroxyisobutyrylated proteins according to biological process. **(B)** Classification of the 2-hydroxyisobutyrylated proteins according to molecular function. **(C)** Classification of the 2-hydroxyisobutyrylated proteins according to cellular component. **(D)** Subcellular localization of the 2-hydroxyisobutyrylated proteins in *F. oxysporum*.

To further explore the function of K_*hib*_ in *F. oxysporum*, we performed a GO-term classification analysis of all identified 2-hydroxyisobutyrylated proteins according to their biological process, molecular function and cellular component. The results of the GO analysis of the 2-hydroxyisobutyrylome indicated that the 2-hydroxyisobutyrylated proteins have a large range of biological processes, molecular functions and cellular components (Supplementary Table 5). Based on the biological process analysis, the most modified proteins were associated with cellular metabolism (13%) and organic substance metabolism (13%) processes (Figure 4A). According to the molecular function classification analysis, the most 2-hydroxyisobutyrylated proteins were involved in organic cyclic compound and heterocyclic compound binding, accounting for 16 and 15% of all 2-hydroxyisobutyrylated proteins, respectively (Figure 4B). As shown in Figure 4C, the results of the cellular component analysis revealed that the most 2-hydroxyisobutyrylated proteins were distributed in intracellular space (21%), intracellular organelles (18%), and membrane-bounded organelles (16%). These GO functional classification results suggest that K_*hib*_ may be related to the diverse molecular functions of modified proteins and may control the different biological processes in diverse cellular components in *F. oxysporum*.

Based on the subcellular localization prediction analysis of the 2-hydroxyisobutyrylated proteins in *F. oxysporum*, most proteins were located in the cytoplasm (32.89%) and mitochondria (23.88%) (Figure 4D). Importantly, 27.27% of the 2-hydroxyisobutyrylated proteins were located in the nucleus, and these included histone H3, H2A, H2B, and H4 (Supplementary Table 6), revealing the key PTMs role of K_*hib*_. In addition, other 2-hydroxyisobutyrylated proteins were located in both the cytoplasm and nucleus (5.79%), in the extracellular space (3.29%) and in the plasma membrane (3.22%). A very small percentage of 2-hydroxyisobutyrylated proteins were predicted to localize in the cytoskeleton (2.23%) and other locations (0.74%). These results suggest that 2-hydroxyisobutyrylated proteins have a widespread distribution in *F. oxysporum*.

### Functional Enrichment Analysis

**FIGURE 5:**
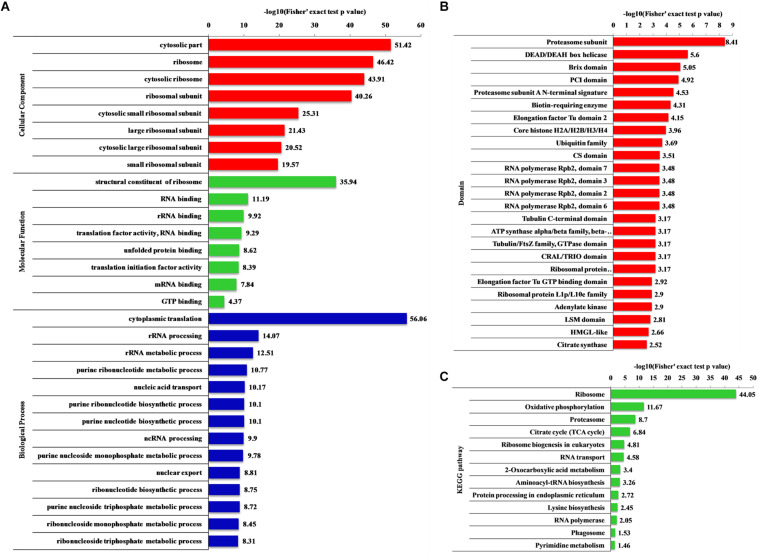
Enrichment analysis of the 2-hydroxyisobutyrylated proteins in *Fusarium oxysporum*. **(A)** GO-based enrichment analysis in terms of biological process (blue), molecular function (green) and cell component (red). **(B)** Domain-based enrichment analysis of the 2-hydroxyisobutyrylated proteins. **(C)** KEGG pathway-based enrichment analysis of the 2-hydroxyisobutyrylated proteins. The value of –log10 (Fisher’s test *p* value) is shown on right of the columns.

Gene ontology (biological processes, molecular functions, and cellular components), KEGG pathway and protein domain enrichment analyses were performed to further understand the characteristics of 2-hydroxyisobutyrylated proteins in *F. oxysporum*. In the biological processes category, a large number of 2-hydroxyisobutyrylated proteins were mainly enriched in cytoplasmic translation, metabolic and biosynthetic processes, indicating that 2-hydroxyisobutyrylated proteins may have a potential function in protein biosynthesis and processing (Figure 5A and Supplementary Tables 7–9). Consistent with these findings, an enrichment analysis of the molecular functions suggested that these modified proteins were mainly involved in the structural constituent of ribosomes and in binding and translation activities. In the cellular compound enrichment analysis, most of the proteins were enriched in the cytosol and ribosome.

The KEGG pathway enrichment analysis revealed that most 2-hydroxyisobutyrylated proteins were significantly enriched in 13 pathways. In agreement with the GO enrichment analysis, the ribosome pathway (map03010), which is a highly conserved pathway, was the significantly enriched pathway, suggesting an important role of K_*hib*_ in protein biosynthesis. Remarkably, several energy production-related pathways were also enriched, and these included oxidative phosphorylation (map00190), the TCA cycle (map00020) and 2-oxocarboxylic acid metabolism (map012102). Based on the pathway enrichment analysis, the 2-hydroxyisobutyrylated proteins of *S. cerevisiae* were also enriched in the ribosome pathway and some metabolic pathways, and these results are consistent with findings in this study (Figure 5C and Supplementary Table 10).

The enrichment analysis of the protein domain demonstrated that the proteasome subunit, RNA polymerase Rpb2, elongation factor Tu GTP-binding and LSM domains were all enriched and tended to be 2-hydroxyisobutyrylated in *F. oxysporum* proteins (Figure 5B and Supplementary Table 11). Taken together, these results showed that 2-hydroxyisobutyrylated proteins are widely distributed in cells and associated with diverse pathways, suggesting that K_*hib*_ plays an important role in cell metabolism and amino acid biosynthesis.

### Protein–Protein Interaction Network of 2-Hydroxyisobutyrylated Proteins in *F. oxysporum*

**FIGURE 6:**
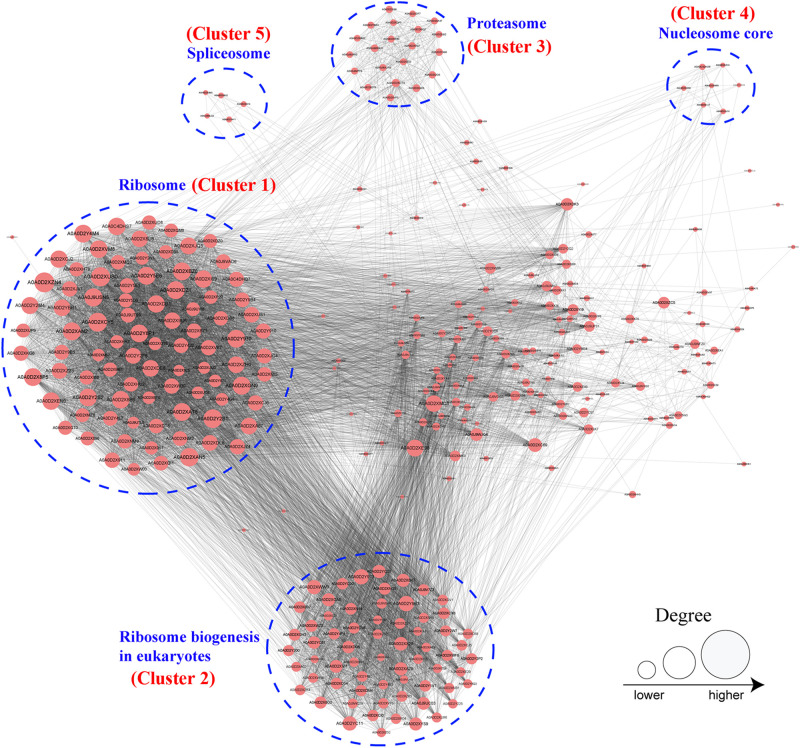
Analysis of protein–protein interaction networks of the 2-hydroxyisobutyrylated proteins in *Fusarium oxysporum*. The top five clusters were shown in blue dotted circle. And the size of the circle indicated number of 2-hydroxyisobutyrylated sites.

To further investigate the cellular processes regulated by K_*hib*_ in *F. oxysporum*, the protein–protein interaction network was established using the STRING database. The interaction network from STRING was visualized in the Cytoscape program (Supplementary Table 12). The results showed that 325 2-hydroxyisobutyrylated proteins were mapped to the protein–protein interaction network, which presents how protein 2-hydroxyisobutyrylation performs diverse pathways in *F. oxysporum*. According to the Cytoscape program, nine highly interconnected clusters of 2-hydroxyisobutyrylated proteins were retrieved, and the top five identified clusters (clusters 1–5) included proteins associated with ribosome, ribosome biogenesis in eukaryotes, proteasome, nucleosome core, and spliceosome (Figure 6 and Supplementary Figure 2). The greater the degree is, the more proteins it interacts with, revealing that the protein is more important in the interaction network. The protein–protein interaction network demonstrated that K_*hib*_ was related to the ribosome, proteasome, and spliceosome and regulated a variety of pathways in *F. oxysporum*.

### Analysis of 2-Hydroxyisobutyrylated Proteins Involved in Virulence and Conidiation in *F. oxysporum*

In this study, we found that several 2-hydroxyisobutyrylated proteins were involved in the virulence and conidiation of *F. oxysporum* (Table 1), including a G protein beta subunit (FoFGB1), which was implicated in cell growth, conidiation, and virulence (Jain et al., 2003). Among these identified proteins, FoFmk1 and FoHog1, two MAP kinase signaling proteins, are critical for the virulence of *F. oxysporum* (Pareek and Rajam, 2017). In addition, the GTP-binding protein FoRho1, a key enzyme for cell wall biosynthesis, was also found to be 2-hydroxyisobutyrylated and contains four 2-hydroxyisobutyrylated sites (Martinez-Rocha et al., 2008). FoPtc1, a serine/threonine phosphatase, regulates phosphorylation of the high osmolarity glycerol response (HOG) pathway in response to osmotic stress and is involved in conidiation (Lemos et al., 2018). It was also found that K_*hib*_ occurs in the two-component histidine kinase Fhk1, which is associated with the virulence-related function in *F. oxysporum* (Rispail and Di Pietro, 2010). In conclusion, these results reveal that K_*hib*_ plays an important role in virulence and conidiation in *F. oxysporum*.

**TABLE 1:** The 2-hydroxyisobutyrylated proteins involved in virulence and conidiation of *Fusarium oxysporum.*

**Protein**	**Gene name**	**Annotation**	**Positions**	**Function**	**References**
FoFGB1	FOXG_11532	G protein beta subunit	36	Virulence, conidiation	Jain et al., 2003
FoFmk1	FOXG_08140	CMGC/MAPK protein kinase	193	Virulence	Pareek and Rajam, 2017
FoHog1	FOXG_06318	Mitogen-activated protein kinase	49, 285	Virulence	Pareek and Rajam, 2017
FoRho1	FOXG_13835	GTP-binding protein rhoA	126, 137, 155, 162	Virulence	Martinez-Rocha et al., 2008
FoPtc1	FOXG_11525	Protein phosphatase	356	Conidiation	Lemos et al., 2018
FoFhk1	FOXG_01684	Two-component histidine kinase	602	Virulence	Rispail and Di Pietro, 2010

### The K_*hib*_ Levels in Conidia and Mycelia of *F. oxysporum*

**FIGURE 7:**
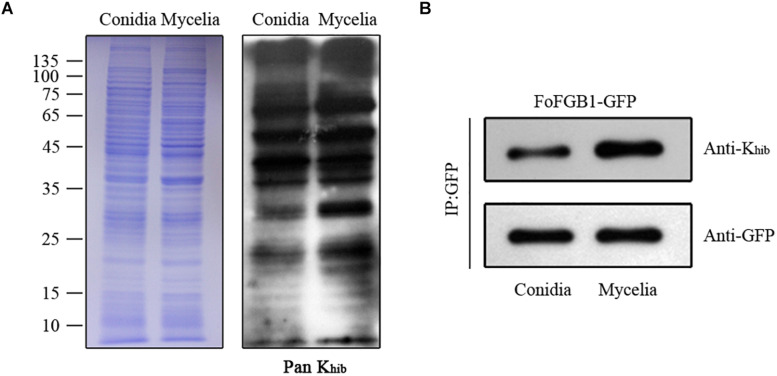
The K_*hib*_ levels of *Fusarium oxysporum* in conidial and mycelial stages. **(A)**. Immunoblot analysis of 2-hydroxyisobutyrylated proteins with pan anti-K_*hib*_ antibody of *F. oxysporum* in conidial and mycelial stages. The loading control by coomassie blue staining was used to ensure that equal amounts of protein were loaded in each lane. **(B)** The FoFGB1-GFP was immunoprecipitated by anti-GFP beads. Anti-GFP and anti-K_*hib*_ antibodies were used to detect FoFGB1-GFP and its K_*hib*_ level, respectively.

To determine the differences in K_*hib*_ levels in various growth stages of *F. oxysporum*, we carried out immunoblotting of 2-hydroxyisobutyrylated proteins in conidia and mycelia. As shown in Figure 7A, a large number of protein bands were observed, and the K_*hib*_ level in mycelia of *F. oxysporum* was higher than that in conidia. Remarkably, the K_*hib*_ level of FoFGB1 was also higher in mycelia than in conidia (Figure 7B). These results suggested that K_*hib*_ occurred in various growth stages in *F. oxysporum*, but the level was higher in the vegetative growth stage. It is worthwhile characterizing the functions and mechanisms of the 2-hydroxyisobutyrylated proteins in different growth stages in future research.

## Discussion

Lysine 2-hydroxyisobutyrylation is one of the most common PTMs in both prokaryotes and eukaryotes, which play key roles in diversified biological processes with multiple functions. First, K_*hib*_ was found in mouse and human histone proteins (Dai et al., 2014; Cheng et al., 2020), and then in *P. patens* (Yu et al., 2017) and rice (Meng et al., 2017) on both histones and non-histones. Although the K_*hib*_ is widely distributed in different species, knowledge about this modification in filamentous fungi is still limited.

In this study, we determined the K_*hib*_ sites in *F. oxysporum* using a specific antibody and high-resolution LC-MS/MS analysis (Supplementary Figure 1A) and a total of 3782 K_*hib*_ sites in 1299 proteins were identified, accounting for about 7% of the *F. oxysporum* proteome. And the western blot assay also suggested that the K_*hib*_ was occurred in *F. oxysporum* (Figure 7). Most proteins contain one K_*hib*_ site, accounting for 43.6% of all 2-hydroxyisobutyrylated proteins, and the sites were distributed in different protein secondary structures in *F. oxysporum* (Figure 1). In addition, the identification of several particular amino acid motifs near K_*hib*_ sites by bioinformatics analysis indicated the substrate preference of K_*hib*_ in *F. oxysporum*. For example, the amino acids D (Aspartic acid) and E (Glutamic acid), which containing the negatively charged side chains, showed a strong bias around the positions of K_*hib*_ in *F. oxysporum* (Figure 2). In the rice seed, the amino acids D and E have a trendency to be located around the K_*hib*_ sites (Meng et al., 2017). The negative charge amino acids, D and E, were also strongly preferred around the 2-hydroxyisobutyrylated sites in the *P. patens* (Vidali and Bezanilla, 2012). These results indicated that the position of lysine and the amino acid around the site plays important roles in K_*hib*_ modification.

In order to confirm the conservation of the K_*hib*_, we have searched against the five organisms with 2-hydroxyisobutyrylated protein sequences in *F. oxysporum*, the results showed that the K_*hib*_ is distributed widely and conserved in a variety of organisms. Furthermore, the protein–protein interaction network analysis suggested that a large range of protein interactions were regulated by K_*hib*_. Functional classification analysis showed that 2-hydroxyisobutyrylated proteins were distributed in almost all part of cellular component and involved in various biological processes in *F. oxysporum* (Figure 4). GO and KEGG pathway enrichment analyses showed that most 2-hydroxyisobutyrylated proteins were involved in the ribosome and oxidative phosphorylation pathways (Supplementary Figure 3), which are critical in living organisms or cells. It is suggested that the K_*hib*_ was related to multiple biological processes and molecular functions in *F. oxysporum*.

Lysine 2-hydroxyisobutyrylation levels were both highly abundant in conidia and mycelia of *F. oxysporum*, but differences still occurred in the two different growth stages. The results of a comparison of conidia and mycelia indicated that most 2-hydroxyisobutyrylated proteins and sites were specific for each stage. The K_*hib*_ level in the mycelial stage was higher than that in the conidial stage, and the different concentrations of substrate and 2-hydroxyisobutyryl-CoA in different stages may contribute to difference in K_*hib*_ abundance. To elucidate whether the different K_*hib*_ levels in the two stages were due to differences in protein abundance, FoFGB1 (with GFP tag), a G protein beta subunit that is important in development and conidia formation, was extracted and purified from conidia and mycelia. Figure 7B shows that the K_*hib*_ level of FoFGB1 was highly significant in the mycelial stage and was not associated with protein abundance. In *Trichophyton rubrum*, the concentration of propionyl-CoA affects the differences in propionylation levels in the conidial and mycelial stages (Xu et al., 2019). In *F. oxysporum*, the concentration of 2-hydroxyisobutyryl-CoA may contribute to the different K_*hib*_ levels between the conidial and mycelial stages.

When fungal pathogens invade host plant tissues, the maintenance of the cell wall integrity is essential for the host root penetration and virulence (Di Pietro et al., 2001; Li et al., 2014). Moreover, several proteins related to the cell wall integrity and virulence of *F. oxysporum* was found to be 2-hydroxyisobutyrylated in this study. Rho-type GTPases could control the expression of cell wall biosynthesis genes through signaling pathways (Levin, 2005). FoRhoI, containing four K_*hib*_ sites, function as cell wall biosynthesis, and the absence of the gene could reduce the virulence of *F. oxysporum* (Table 1; Martinez-Rocha et al., 2008). In some plant fungal pathogens, such as *Botrytis cinerea* (Zheng et al., 2000), *Colletotrichum lagenarium* (Takano et al., 2000), and *Claviceps purpurea* (Mey et al., 2002), the genes of mitogen-activated protein kinase (MAPK) pathway are essential for pathogenicity (Hamel et al., 2012). In this study, we identified several 2-hydroxyisobutyrylated proteins related to the virulence of *F. oxysporum*, including two components of the MAPK pathway: FoFmk1 (K193) and FoHog1 (K49, K285) (Pareek and Rajam, 2017). In the previous research, the deletion of the *fmk1* gene resulted in loss of virulence in tomato plants but no significant difference in conidiation. These results indicated that K_*hib*_ of FoFmk1 may play a key role in virulence of *F. oxysporum*. In addition, the phosphatase Ptc1, which contains one K_*hib*_ site in *F. oxysporum*, responses to stress depend on the regulation of two MAPK pathways (Lemos et al., 2018). Apart from the abovementioned 2-hydroxyisobutyrylated protein, there are other identified proteins were enriched in the MAPK pathway (Supplementary Table 10). Although additional experiments are needed to explain the relationship between K_*hib*_ and the biological functions of *F. oxysporum*, these findings provide some clues and ideas for the exploration of K_*hib*_.

Acyltransferases and deacylases regulate the reversible rection of each PTM *in vivo*, such as KATs and KDACs, which are responsible for acetylation. KATs could transfer the acetyl group to the lysine residue and KDACs have the ability to remove the acetyl group from lysine (Lv et al., 2016). Recent studies have shown that KATs and KDACs have the same function on 2-hydroxyisobutyrylation as acetylation, and KATs can catale the K_*hib*_ and KDACs to remove K_*hib*_. In budding yeast, *S. cerevisiae* and humans (Sapountzi et al., 2006), HAT has K_*hib*_ transferase activity both *in vitro* and *in vivo*. HDAC2 and HDAC3 can catalyze de-2-hydroxyisobutyrylation reactions *in vitro* and *in vivo* in mammalian cells (Huang et al., 2018). Further studies are needed to verify the acyltransferases and deacylases of 2-hydroxyisobutyrylated proteins in *F. oxysporum* and offer a rich source for studying the roles of 2-hydroxyisobutyrylation in different biological processes.

*Fusarium oxysporum* is a soil-borne fungal pathogen and could cause the wilt diseases in more than 150 different plants, including many important crops and trees (Mohali, 1996; Pietro et al., 2003; Perez-Nadales and Di Pietro, 2011; Alwahshi et al., 2019; Lan et al., 2020), that lead to severe losses of production. Integrated disease management (IDM) which is a disease control approach containing the chemical, biological and genetic strategies should be used to control the diseases caused by *F. oxysporum* (Saeed et al., 2017; Kamil et al., 2018). In this study, we found that some 2-hydroxyisobutyrylated proteins were involved in infection and pathogenicity processes of *F. oxysporum* (Table 1). Therefore, identification and analysis of virulence associated proteins which occurring K_*hib*_ modification will be help to widen the comprehensive views of K_*hib*_ in *F. oxysporum* and open up principally new possibilities for disease control in the field.

## Conclusion

Our study is the first report of K_*hib*_ in *F. oxysporum* and provides a resource for further exploration of the potential functions of K_*hib*_ in plant pathogenic fungi. This finding provides some insights into the function of K_*hib*_ in several processes of *F. oxysporum* and detects the abundance of K_*hib*_ in conidial and mycelial stages. This study will improve our comprehension of K_*hib*_ in conidiation and virulence of *F. oxysporum* and other fungal plant pathogens. Although K_*hib*_ proteins play important roles in the virulence and conidiation of *F. oxysporum*, further studies are needed to uncover the detailed mechanism.

## Data Availability Statement

The datasets presented in this study can be found in online repositories. The names of the repository/repositories and accession number(s) can be found in the article/Supplementary Material.

## Author Contributions

WL designed the research. HQ and LW performed the research. XM and XY contributed new reagents or analytic tools. HQ, LW, BW, and WL analyzed the data. HQ and WL wrote the manuscript. All authors contributed to the article and approved the submitted version.

## Conflict of Interest

XM and XY were employed by the company Micron Biotechnology Co., Ltd. The remaining authors declare that the research was conducted in the absence of any commercial or financial relationships that could be construed as a potential conflict of interest.

## Footnotes

1 http://www.ebi.ac.uk/GOA/

2 http://www.ebi.ac.uk/interpro/

## Supplementary Material

Supplementary Figure 1 Overview of identification of lysine 2-hydroxyisobutyrylation (K_*hib*_) proteome in *Fusarium oxysporum*. **(A)** Schematic representation of the experimental procedures used in *F. oxysporum*. **(B)** Venn diagram showed the number of K_*hib*_ proteins and overlaps in all three repeated experiments.

Supplementary Figure 2 Interaction network of 2-hydroxyisobutyrylated proteins associated with ribosome, ribosome biogenesis in eukaryotes, proteasome, nucleosome core, and spliceosome in *Fusarium oxysporum*.

Supplementary Figure 3 Significantly enriched KEGG pathways in *Fusarium oxysporum*. 2-hydroxyisobutyrylated proteins involved in **(A)** Ribosome **(B)** Oxidative phosphorylation. The identified 2-hydroxyisobutyrylated proteins were highlighted in red.

Supplementary Table 1 The 2-hydroxyisobutyrylated sites identified in the three experiments in *Fusarium oxysporum*.

Supplementary Table 2 The number of identified 2-hydroxyisobutyrylated sites in *Fusarium oxysporum*.

Supplementary Table 3 Amino acid sequence analysis from the −10 to +10 positions around the 2-hydroxyisobutyrylated lysine in *Fusarium oxysporum*.

Supplementary Table 4 Conservation of lysine 2-hydroxyisobutyrylated proteins in *Fusarium oxysporum*.

Supplementary Table 5 The distribution of proteins of *Fusarium oxysporum* in GO terms.

Supplementary Table 6 Subcellular localizations of the 2-hydroxyisobutyrylation proteins in *Fusarium oxysporum*.

Supplementary Table 7 Protein GO enrichment based on biological process in *Fusarium oxysporum*.

Supplementary Table 8 Protein GO enrichment based on molecular function in *Fusarium oxysporum*.

Supplementary Table 9 Protein GO enrichment based on cellular component in *Fusarium oxysporum*.

Supplementary Table 10 KEGG pathway enrichment analysis in *Fusarium oxysporum*.

Supplementary Table 11 Protein domain enrichment analysis in *Fusarium oxysporum*.

Supplementary Table 12 Node information in protein–protein interaction network in *Fusarium oxysporum*.
