# Supplementary material for: Proteome-Wide Analysis of Lysine 2-Hydroxyisobutyrylated Proteins in Fusarium oxysporum
Source: Front Microbiol. 2021 Feb 10;12:623735. doi: 10.3389/fmicb.2021.623735 (PMC7902869; doi:10.3389/fmicb.2021.623735)

**Proteome-wide analysis of lysine 2-hydroxyisobutyrylated proteins in *Fusarium oxysporum***

Hengwei Qian^2^, Lulu Wang^1^, Xianliang Ma^3^, Xingling Yi^3^, Baoshan Wang^2^, Wenxing Liang^1*^

*^1^College of Plant Health and Medicine, Qingdao Agricultural University, Qingdao, Shandong 266109, China.*

*^2^College of Life Sciences, Shandong Normal University, Jinan, Shandong 250014, China.*

*^3^Micron biotechnology Co. Ltd, Hangzhou, Zhejiang310051, China.*

Figure S1. Overview of identification of lysine 2-hydroxyisobutyrylation (K_hib_) proteome in *F. oxysporum*. (A) Schematic representation of the experimental procedures used in *F. oxysporum*. (B) Venn diagram showed the number of K_hib_ proteins and overlaps in all three repeated experiments.


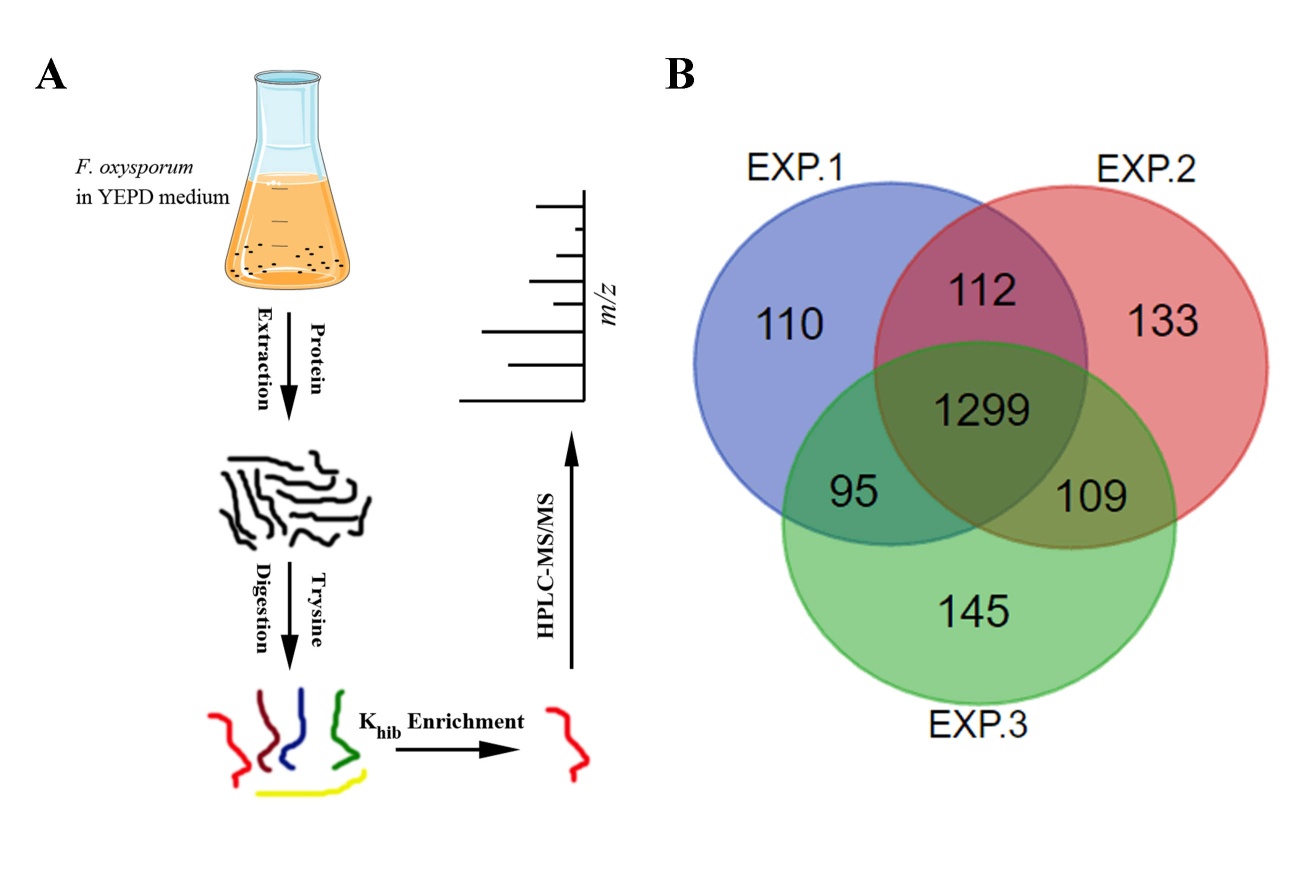


Figure S2. Interaction network of 2-hydroxyisobutyrylated proteins associated with ribosome, ribosome biogenesis in eukaryotes, proteasome, nucleosome core and spliceosome.


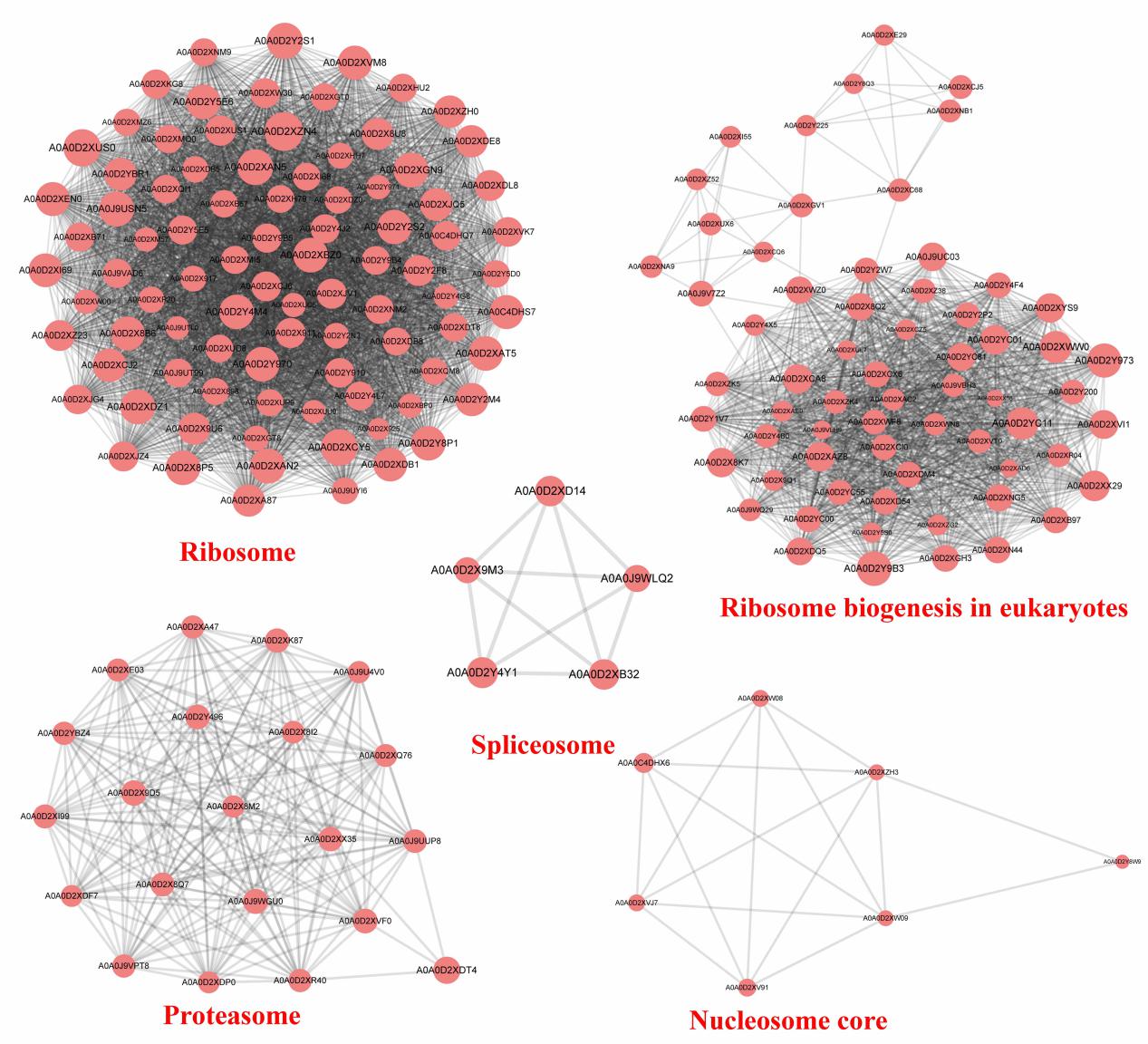


Figure S3. Significantly enriched KEGG pathways. 2-hydroxyisobutyrylated proteins involved in (A) Ribosome (B) Oxidative phosphorylation . The identified 2-hydroxyisobutyrylated proteins were highlighted in red.


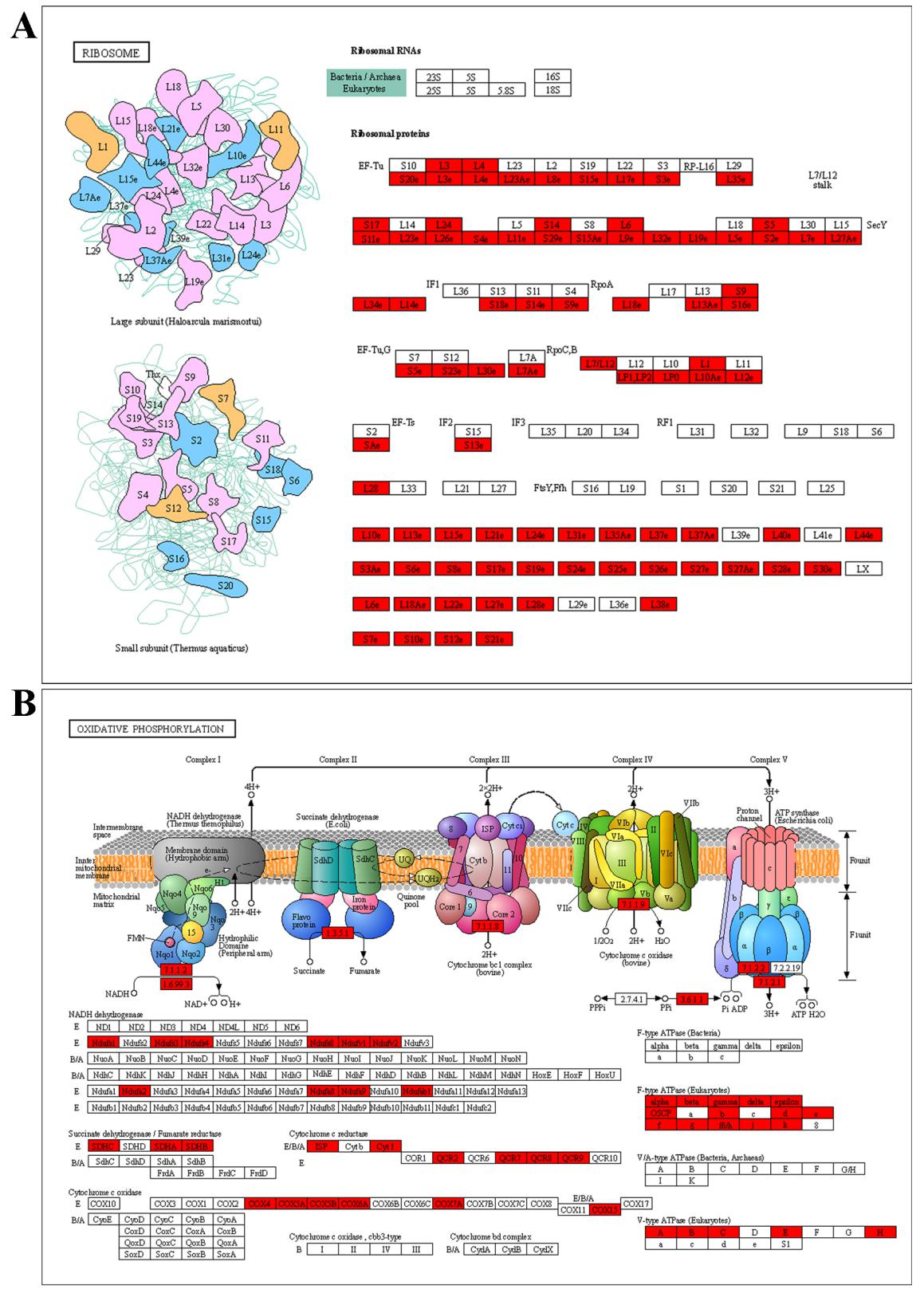

Supplement: Supplementary Figure 1 — Overview of identification of lysine 2-hydroxyisobutyrylation (Khib) proteome in Fusarium oxysporum. (A) Schematic representation of the experimental procedures used in F. oxysporum. (B) Venn diagram showed the number of Khib proteins and overlaps in all three repeated experiments. [file Data_Sheet_1.docx]
